# 3-Methyl-1,5-diphenyl-4,5-dihydro-1*H*-pyrazole

**DOI:** 10.1107/S1600536813007575

**Published:** 2013-03-28

**Authors:** M. Manjula, P. Jayaroopa, B. C. Manjunath, K. Ajay kumar, N. K. Lokanath

**Affiliations:** a Department of Studies in Physics, Manasagangotri, University of Mysore, Mysore 570 006, India; b Post Graduate Department of Chemistry, Yuvaraja’s College, University of Mysore, Mysore 570 006, India

## Abstract

In the title compound, C_16_H_16_N_2_, the dihydro­pyrazole ring adopts a shallow envelope conformation, with the C atom bearing the phenyl group displaced by 0.298 (2) Å from the other atoms (r.m.s. deviation = 0.015 Å). The dihedral angles between the four near coplanar atoms of the central ring and the N- and C-bonded phenyl groups are 13.49 (13) and 82.22 (16)°, respectively.

## Related literature

For background to pyrazoles, see: Govindaraju *et al.* (2012 ▶); Jayaroopa *et al.* (2013 ▶); Kalirajan *et al.* (2013 ▶); Mariappan *et al.* (2010 ▶); Shyama *et al.* (2009 ▶). For related structures, see: Baktır *et al.* (2011 ▶); Fun *et al.* (2011 ▶). 
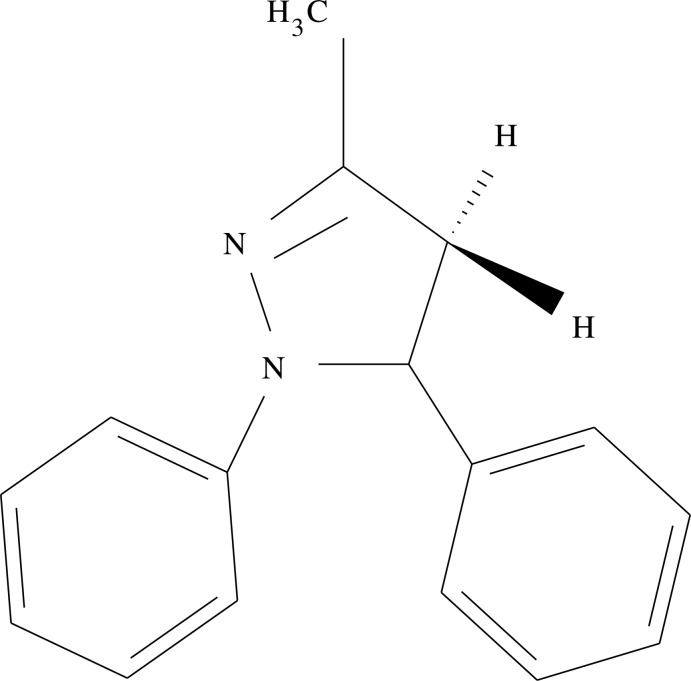


## Experimental

### 

#### Crystal data

C_16_H_16_N_2_
*M* *_r_* = 236.31
Monoclinic, 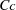

*a* = 18.1224 (17) Å
*b* = 7.8055 (6) Å
*c* = 12.5057 (13) Å
β = 132.207 (9)°
*V* = 1310.3 (3) Å^3^
*Z* = 4
Mo *K*α radiation
μ = 0.07 mm^−1^
*T* = 301 K
0.32 × 0.20 × 0.18 mm

#### Data collection

Oxford Diffraction Xcalibur Eos diffractometer
11856 measured reflections
2973 independent reflections
2362 reflections with *I* > 2σ(*I*)
*R*_int_ = 0.033

#### Refinement

*R*[*F* ^2^ > 2σ(*F* ^2^)] = 0.037
*wR*(*F* ^2^) = 0.131
*S* = 0.91
2973 reflections
165 parameters
2 restraints
H-atom parameters constrained
Δρ_max_ = 0.14 e Å^−3^
Δρ_min_ = −0.10 e Å^−3^

### 

Data collection: *CrysAlis PRO* (Oxford Diffraction, 2009 ▶); cell refinement: *CrysAlis PRO*; data reduction: *CrysAlis PRO*; program(s) used to solve structure: *SHELXS97* (Sheldrick, 2008 ▶); program(s) used to refine structure: *SHELXL97* (Sheldrick, 2008 ▶); molecular graphics: *Mercury* (Macrae *et al.*, 2006 ▶); software used to prepare material for publication: *Mercury*.

## Supplementary Material

Crystal structure: contains datablock(s) global, I. DOI: 10.1107/S1600536813007575/hb7053sup1.cif

Structure factors: contains datablock(s) I. DOI: 10.1107/S1600536813007575/hb7053Isup2.hkl

Supplementary material file. DOI: 10.1107/S1600536813007575/hb7053Isup3.cml

Additional supplementary materials:  crystallographic information; 3D view; checkCIF report

## Figures and Tables

**Fig. 1.**
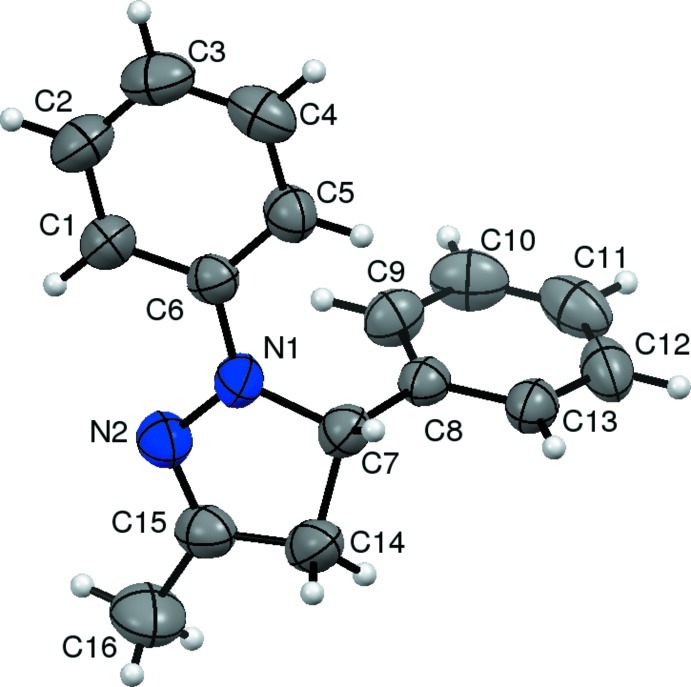
ORTEP diagram of the title molecule with 50% probability ellipsoids.

**Fig. 2.**
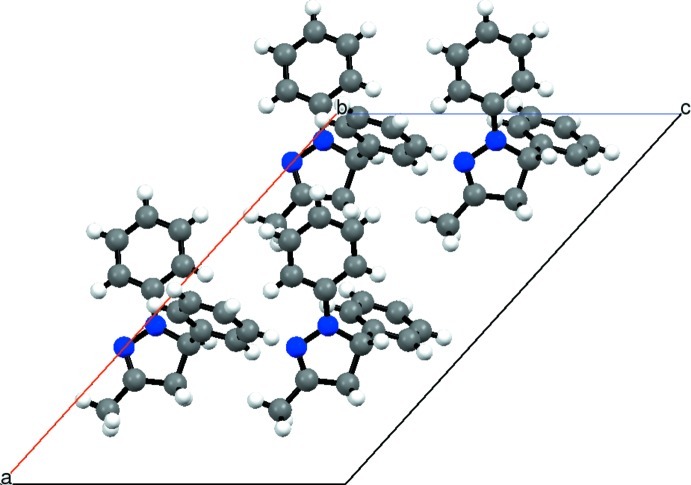
Packing diagram of molecule, viewed along the crystallographic b axis.

## supplementary crystallographic information

### Comment

Pyrazoles are five-member heterocycles with two nitrogen atoms in a ring at 1,2-positions. They have been efficiently transformed in to a potential medicinally and pharmaceutically important molecule. Pyrazole derivatives have known to exhibit diverse biological applications such as antidiabetic, anaesthetic, antifungal (Jayaroopa *et al.*, 2013), antiandrogenic, antioxidant, analgesic and anti-inflammatory activities. In addition, they have also showed potential anti-bacterial (Govindaraju *et al.*, 2012), anticancer (Kalirajan *et al.*, 2013), antiamoebic, potent and selective inhibitors of tissue-nonspecific alkaline phosphatase (Shyama *et al.*, 2009), anti-inflammatory and protein kinase C inhibitor (Mariappan *et al.*, 2010) properties.

we have synthesized the title compound to study its crystal structure in order to understand the structure-activity relationship for its biological activity.

The title compound C_16_ H_16_ N_2_, contains two benzene rings (C1-C6 and C8-C13), these two rings are attached to the central pyrazole ring and the molecules are connected by non classical hydrogen bonds. The dihydropyrazole ring is a shallow envelope, with atom C7 displaced from the other four atoms by 0.298 (2)Å. The dihedral angles between the four near coplanar atoms of the central ring and the N- and C-bonded phenyl groups are 13.49 (13) and 82.22 (16)°, respectively. Bond lengths and bond angles are within normal ranges and are comparable to related structures (Baktır *et al.*, 2011 & Fun *et al.*, 2011).

### Experimental

A mixture of 4-phenylbut-3-en-2-one (0.05 mmol), phenyl hydrazine hydrochloride (0.05 mmol) and sodium acetate (0.05 mmol) in ethyl alcohol (25 ml) was stirred at room temperature for 1 h. The progress of the reaction was monitored by TLC. After the completion of the reaction, the mixture was poured into ice cold water. The solid formed was separated and crystallized with acetonitrile to get the title compound as yellow blocks.

Flash Point: 178 ° C. ^1^H NMR (CDCl3): δ 2.015 (s, 3H, CH3), 2.619–2.671 (q, 1H, C4—H), 3.475–3.536 (q, 1H, C4—H), 5.108–5.146 (q, 1H, C5—H), 6.622–6.655 (t, 1H, Ar—H), 6.805–6.829 (d, 2H, Ar—H), 7.061–7.097 (t, 2H, Ar—H), 7.230–7.373 (m, 5H, Ar—H).

### Refinement

All hydrogen atoms were located geometrically with C—H = 0.93–0.97) Å and allowed to ride on their parent atoms with *U*_iso_(H) = 1.2*U*_eq_(aromatic C).

### Crystal data

**Table:**

C_16_H_16_N_2_	*F*(000) = 504
*M**_r_* = 236.31	*D*_x_ = 1.198 Mg m^−^^3^
Monoclinic, *C**c*	Melting point = 363–365 K
Hall symbol: C -2yc	Mo *K*α radiation, λ = 0.71073 Å
*a* = 18.1224 (17) Å	Cell parameters from 2973 reflections
*b* = 7.8055 (6) Å	θ = 3.0–27.6°
*c* = 12.5057 (13) Å	µ = 0.07 mm^−^^1^
β = 132.207 (9)°	*T* = 301 K
*V* = 1310.3 (3) Å^3^	Block, pale yellow
*Z* = 4	0.32 × 0.20 × 0.18 mm

### Data collection

**Table:**

Oxford Diffraction Xcalibur Eos diffractometer	2362 reflections with *I* > 2σ(*I*)
Radiation source: fine-focus sealed tube	*R*_int_ = 0.033
Graphite monochromator	θ_max_ = 27.6°, θ_min_ = 3.0°
Detector resolution: 16.0839 pixels mm^-1^	*h* = −23→23
ω scans	*k* = −10→10
11856 measured reflections	*l* = −16→16
2973 independent reflections	

### Refinement

**Table:**

Refinement on *F*^2^	Primary atom site location: structure-invariant direct methods
Least-squares matrix: full	Secondary atom site location: difference Fourier map
*R*[*F*^2^ > 2σ(*F*^2^)] = 0.037	Hydrogen site location: inferred from neighbouring sites
*wR*(*F*^2^) = 0.131	H-atom parameters constrained
*S* = 0.91	*w* = 1/[σ^2^(*F*_o_^2^) + (0.1*P*)^2^] where *P* = (*F*_o_^2^ + 2*F*_c_^2^)/3
2973 reflections	(Δ/σ)_max_ = 0.001
165 parameters	Δρ_max_ = 0.14 e Å^−^^3^
2 restraints	Δρ_min_ = −0.10 e Å^−^^3^

### Special details

**Table:**

Geometry. Bond distances, angles *etc*. have been calculated using the rounded fractional coordinates. All su's are estimated from the variances of the (full) variance-covariance matrix. The cell e.s.d.'s are taken into account in the estimation of distances, angles and torsion angles
Refinement. Refinement on *F*^2^ for ALL reflections except those flagged by the user for potential systematic errors. Weighted *R*-factors *wR* and all goodnesses of fit *S* are based on *F*^2^, conventional *R*-factors *R* are based on *F*, with *F* set to zero for negative *F*^2^. The observed criterion of *F*^2^ > σ(*F*^2^) is used only for calculating -*R*-factor-obs *etc*. and is not relevant to the choice of reflections for refinement. *R*-factors based on *F*^2^ are statistically about twice as large as those based on *F*, and *R*-factors based on ALL data will be even larger.

### Fractional atomic coordinates and isotropic or equivalent isotropic displacement parameters (Å^2^)

**Table:**

	*x*	*y*	*z*	*U*_iso_*/*U*_eq_	
N1	0.57189 (12)	0.5350 (3)	0.53221 (17)	0.0583 (6)	
N2	0.63012 (12)	0.5538 (2)	0.49747 (18)	0.0528 (5)	
C1	0.43700 (15)	0.4218 (3)	0.2954 (2)	0.0539 (6)	
C2	0.34060 (17)	0.3632 (3)	0.1948 (2)	0.0683 (7)	
C3	0.27852 (16)	0.3631 (3)	0.2214 (3)	0.0694 (7)	
C4	0.31410 (16)	0.4245 (3)	0.3516 (3)	0.0617 (7)	
C5	0.41076 (14)	0.4857 (2)	0.4546 (2)	0.0529 (6)	
C6	0.47391 (13)	0.4829 (2)	0.42818 (19)	0.0439 (5)	
C7	0.62148 (15)	0.5986 (2)	0.6767 (2)	0.0487 (5)	
C8	0.58591 (12)	0.7740 (2)	0.67789 (18)	0.0425 (5)	
C9	0.53469 (16)	0.8844 (3)	0.5615 (2)	0.0558 (6)	
C10	0.50989 (18)	1.0475 (3)	0.5715 (3)	0.0710 (8)	
C11	0.53636 (18)	1.1018 (3)	0.6979 (3)	0.0725 (9)	
C12	0.58650 (17)	0.9929 (3)	0.8138 (3)	0.0644 (8)	
C13	0.61057 (14)	0.8294 (3)	0.80360 (19)	0.0502 (6)	
C14	0.73007 (16)	0.6028 (3)	0.7433 (2)	0.0618 (6)	
C15	0.71843 (15)	0.5947 (2)	0.6134 (2)	0.0563 (6)	
C16	0.8000 (2)	0.6260 (4)	0.6152 (4)	0.0878 (11)	
H1	0.47760	0.42050	0.27480	0.0650*	
H2	0.31700	0.32270	0.10660	0.0820*	
H3	0.21360	0.32240	0.15260	0.0830*	
H4	0.27280	0.42500	0.37110	0.0740*	
H5	0.43330	0.52860	0.54160	0.0640*	
H7	0.61430	0.51580	0.72810	0.0580*	
H9	0.51670	0.84890	0.47570	0.0670*	
H10	0.47520	1.12070	0.49240	0.0850*	
H11	0.52030	1.21200	0.70480	0.0870*	
H12	0.60430	1.02910	0.89940	0.0770*	
H13	0.64380	0.75570	0.88220	0.0600*	
H14A	0.76320	0.70760	0.79730	0.0740*	
H14B	0.76730	0.50530	0.80630	0.0740*	
H16A	0.77370	0.62300	0.51830	0.1320*	
H16B	0.85000	0.53900	0.67170	0.1320*	
H16C	0.82900	0.73640	0.65690	0.1320*	

### Atomic displacement parameters (Å^2^)

**Table:**

	*U*^11^	*U*^22^	*U*^33^	*U*^12^	*U*^13^	*U*^23^
N1	0.0465 (9)	0.0796 (11)	0.0514 (9)	−0.0147 (8)	0.0339 (8)	−0.0253 (8)
N2	0.0518 (9)	0.0527 (8)	0.0596 (9)	−0.0064 (7)	0.0397 (8)	−0.0083 (7)
C1	0.0477 (11)	0.0618 (11)	0.0449 (9)	0.0008 (8)	0.0281 (9)	−0.0037 (9)
C2	0.0521 (12)	0.0800 (14)	0.0474 (11)	−0.0079 (10)	0.0231 (10)	−0.0109 (10)
C3	0.0457 (11)	0.0665 (13)	0.0668 (14)	−0.0070 (9)	0.0259 (11)	−0.0034 (10)
C4	0.0532 (11)	0.0519 (10)	0.0866 (15)	0.0036 (9)	0.0497 (11)	0.0054 (10)
C5	0.0551 (11)	0.0494 (10)	0.0590 (11)	−0.0021 (8)	0.0403 (10)	−0.0077 (8)
C6	0.0413 (9)	0.0383 (7)	0.0461 (9)	0.0022 (7)	0.0269 (8)	−0.0015 (7)
C7	0.0479 (9)	0.0476 (9)	0.0425 (9)	−0.0016 (7)	0.0271 (8)	−0.0040 (7)
C8	0.0391 (8)	0.0454 (8)	0.0420 (8)	−0.0051 (7)	0.0269 (7)	−0.0026 (7)
C9	0.0574 (11)	0.0567 (11)	0.0491 (10)	0.0004 (8)	0.0341 (9)	0.0071 (8)
C10	0.0650 (14)	0.0544 (11)	0.0800 (16)	0.0095 (10)	0.0431 (13)	0.0199 (11)
C11	0.0664 (14)	0.0499 (12)	0.110 (2)	0.0015 (9)	0.0628 (15)	−0.0042 (12)
C12	0.0686 (14)	0.0652 (13)	0.0789 (15)	−0.0099 (10)	0.0575 (13)	−0.0201 (11)
C13	0.0490 (10)	0.0567 (10)	0.0457 (10)	−0.0039 (8)	0.0322 (9)	−0.0032 (8)
C14	0.0480 (11)	0.0595 (11)	0.0545 (11)	0.0037 (9)	0.0249 (9)	−0.0119 (9)
C15	0.0463 (11)	0.0505 (10)	0.0644 (12)	−0.0024 (8)	0.0340 (10)	−0.0060 (9)
C16	0.0631 (15)	0.105 (2)	0.100 (2)	−0.0193 (14)	0.0567 (15)	−0.0180 (16)

### Geometric parameters (Å, º)

**Table:**

N1—N2	1.393 (4)	C14—C15	1.492 (3)
N1—C6	1.381 (3)	C15—C16	1.484 (6)
N1—C7	1.462 (3)	C1—H1	0.9300
N2—C15	1.284 (3)	C2—H2	0.9300
C1—C2	1.375 (4)	C3—H3	0.9300
C1—C6	1.393 (3)	C4—H4	0.9300
C2—C3	1.371 (5)	C5—H5	0.9300
C3—C4	1.373 (4)	C7—H7	0.9800
C4—C5	1.387 (4)	C9—H9	0.9300
C5—C6	1.388 (4)	C10—H10	0.9300
C7—C8	1.518 (3)	C11—H11	0.9300
C7—C14	1.538 (4)	C12—H12	0.9300
C8—C9	1.381 (3)	C13—H13	0.9300
C8—C13	1.384 (3)	C14—H14A	0.9700
C9—C10	1.383 (4)	C14—H14B	0.9700
C10—C11	1.375 (4)	C16—H16A	0.9600
C11—C12	1.371 (4)	C16—H16B	0.9600
C12—C13	1.382 (4)	C16—H16C	0.9600
			
N2—N1—C6	120.09 (17)	C2—C3—H3	121.00
N2—N1—C7	112.6 (2)	C4—C3—H3	121.00
C6—N1—C7	126.7 (2)	C3—C4—H4	120.00
N1—N2—C15	107.8 (2)	C5—C4—H4	119.00
C2—C1—C6	120.1 (3)	C4—C5—H5	120.00
C1—C2—C3	121.6 (2)	C6—C5—H5	120.00
C2—C3—C4	118.6 (3)	N1—C7—H7	110.00
C3—C4—C5	121.0 (3)	C8—C7—H7	110.00
C4—C5—C6	120.2 (2)	C14—C7—H7	110.00
N1—C6—C1	120.5 (3)	C8—C9—H9	120.00
N1—C6—C5	121.00 (18)	C10—C9—H9	120.00
C1—C6—C5	118.5 (2)	C9—C10—H10	120.00
N1—C7—C8	113.95 (16)	C11—C10—H10	120.00
N1—C7—C14	100.2 (2)	C10—C11—H11	120.00
C8—C7—C14	111.89 (18)	C12—C11—H11	120.00
C7—C8—C9	122.80 (19)	C11—C12—H12	120.00
C7—C8—C13	118.56 (16)	C13—C12—H12	120.00
C9—C8—C13	118.55 (19)	C8—C13—H13	120.00
C8—C9—C10	120.5 (2)	C12—C13—H13	120.00
C9—C10—C11	120.3 (2)	C7—C14—H14A	111.00
C10—C11—C12	119.7 (2)	C7—C14—H14B	111.00
C11—C12—C13	120.1 (3)	C15—C14—H14A	111.00
C8—C13—C12	120.8 (2)	C15—C14—H14B	111.00
C7—C14—C15	102.47 (18)	H14A—C14—H14B	109.00
N2—C15—C14	113.4 (3)	C15—C16—H16A	109.00
N2—C15—C16	122.1 (2)	C15—C16—H16B	109.00
C14—C15—C16	124.5 (2)	C15—C16—H16C	109.00
C2—C1—H1	120.00	H16A—C16—H16B	110.00
C6—C1—H1	120.00	H16A—C16—H16C	110.00
C1—C2—H2	119.00	H16B—C16—H16C	109.00
C3—C2—H2	119.00		
			
C6—N1—N2—C15	−176.91 (18)	C4—C5—C6—N1	−176.63 (19)
C7—N1—N2—C15	11.2 (2)	C4—C5—C6—C1	1.7 (3)
N2—N1—C6—C1	10.6 (3)	N1—C7—C8—C9	−18.0 (4)
N2—N1—C6—C5	−171.14 (17)	N1—C7—C8—C13	165.6 (2)
C7—N1—C6—C1	−178.8 (2)	C14—C7—C8—C9	94.8 (3)
C7—N1—C6—C5	−0.5 (3)	C14—C7—C8—C13	−81.6 (3)
N2—N1—C7—C8	101.5 (3)	N1—C7—C14—C15	17.20 (19)
N2—N1—C7—C14	−18.2 (2)	C8—C7—C14—C15	−103.93 (19)
C6—N1—C7—C8	−69.7 (3)	C7—C8—C9—C10	−175.5 (3)
C6—N1—C7—C14	170.6 (2)	C13—C8—C9—C10	0.8 (4)
N1—N2—C15—C14	1.8 (2)	C7—C8—C13—C12	175.1 (3)
N1—N2—C15—C16	−179.6 (2)	C9—C8—C13—C12	−1.4 (4)
C6—C1—C2—C3	0.1 (3)	C8—C9—C10—C11	0.3 (5)
C2—C1—C6—N1	177.2 (2)	C9—C10—C11—C12	−0.8 (6)
C2—C1—C6—C5	−1.1 (3)	C10—C11—C12—C13	0.2 (6)
C1—C2—C3—C4	0.5 (4)	C11—C12—C13—C8	0.9 (5)
C2—C3—C4—C5	0.1 (4)	C7—C14—C15—N2	−12.9 (2)
C3—C4—C5—C6	−1.1 (3)	C7—C14—C15—C16	168.6 (2)
